# Efficacy of the unified protocol for the treatment of emotional disorders in the Spanish public mental health system using a group format: study protocol for a multicenter, randomized, non-inferiority controlled trial

**DOI:** 10.1186/s12955-018-0866-2

**Published:** 2018-03-12

**Authors:** Jorge Osma, Carlos Suso-Ribera, Azucena García-Palacios, Elena Crespo-Delgado, Cristina Robert-Flor, Ana Sánchez-Guerrero, Vanesa Ferreres-Galan, Luisa Pérez-Ayerra, Amparo Malea-Fernández, Mª Ángeles Torres-Alfosea

**Affiliations:** 1 University of Zaragoza, Teruel, Spain; 2 Instituto de Investigación Sanitaria de Aragón, Zaragoza, Spain; 3 Universitat Jaume I. Edificio de Investigación II, Av. Vicente SosBaynat s/n, 12006 Castellón, Spain; 4 Unidad de Salud Mental La Fuente de San Luís, C/ Arabista Ambrosio Huici, 30, 46013 Valencia, Spain; 5 Hospital Comarcal de Vinaròs, Avinguda Gil d’Atrosillo, s/n, 12500 Castellón, Spain; 6 Centro de Salud Mental La Milagrosa, C/ Julián Gayarre, 1A, 31005 Pamplona, Spain; 7 Centro de Salud Mental La Malvarrosa, Av. Malva-Rosa, 10, 46011 Valencia, Spain; 8 Hospital General Universitario de Alicante, C/ Pintor Baeza, 11, 03010 Alicante, Spain; 9 Facultad de Ciencias Sociales y Humanas, Universidad de Zaragoza, C/ Ciudad Escolar s/n, 44003 Teruel, Spain; 10 CIBER of Physiopathology of Obesity and Nutrition CIBERobn, CB06/03 Instituto de Salud Carlos III, Castellón, Spain

**Keywords:** Unified protocol, Transdiagnostic, Emotional disorders, Group therapy, Public mental health, Multicenter, Randomized, Controlled trial

## Abstract

**Background:**

Emotional disorders, which include both anxiety and depressive disorders, are the most prevalent psychological disorders according to recent epidemiological studies. Consequently, public costs associated with their treatment have become a matter of concern for public health systems, which face long waiting lists. Because of their high prevalence in the population, finding an effective treatment for emotional disorders has become a key goal of today’s clinical psychology. The Unified Protocol for the Transdiagnostic Treatment of Emotional Disorders might serve the aforementioned purpose, as it can be applied to a variety of disorders simultaneously and it can be easily performed in a group format.

**Methods:**

The study is a multicenter, randomized, non-inferiority controlled clinical trial. Participants will be 220 individuals with emotional disorders, who are randomized to either a treatment as usual (individual cognitive behavioral therapy) or to a Unified Protocol condition in group format. Depression, anxiety, and diagnostic criteria are the primary outcome measures. Secondary measures include the assessment of positive and negative affect, anxiety control, personality traits, overall adjustment, and quality of life. An analysis of treatment satisfaction is also conducted. Assessment points include baseline, post-treatment, and three follow-ups at 3, 6, and 12 months. To control for missing data and possible biases, intention-to-treat and per-protocol analyses will be performed.

**Discussion:**

This is the first randomized, controlled clinical trial to test the effectiveness of a transdiagnostic intervention in a group format for the treatment of emotional disorders in public settings in Spain. Results obtained from this study may have important clinical, social, and economic implications for public mental health settings in Spain.

**Trial registration:**

Retrospectively registered at https://clinicaltrials.gov/. Trial NCT03064477 (March 10, 2017). The trial is active and recruitment is ongoing. Recruitment is expected to finish by January 2020.

## Background

Emotional disorders (EDs; i.e., depressive and anxiety disorders) have become the most prevalent psychiatric disorders globally [1]. The 12-month prevalence of anxiety and depressive disorders affect 14% and 7.8% (6.9% by major depression) of the population, respectively [2], and comordibity may be as high as 50% [3]. Lifetime prevalence rates in primary care settings in Spain reveal that mood and anxiety disorders, as defined in the DSM-IV-TR) [4], are the most prevalent psychiatric problems as well, with 35.8% and 25.6%, respectively [5].

As a result of their high prevalence in the population, EDs have become a global health problem due to their associated costs. For instance, a study conducted in 36 countries estimated that the annual treatment cost of depressive disorders and anxiety problems amounted to $91 billion and $56 billion, respectively [6]. It has been calculated that more than 12 billion days of productivity loss are attributable to depression and anxiety disorders every year, which results in $925 billion in productivity losses. In Spain, when direct and indirect costs are included, mood and anxiety disorders (again, according to DMS-IV-TR categories), are estimated to cost approximately $10.76 billion and $10.36 billion, respectively [7]. The high prevalence and important burden of EDs indicate that there is an urgent need to enhance the effectiveness of treatments for EDs in public mental health systems.

The Unified Protocol (UP) for the Treatment of Emotional Disorders [8, 9] is a recently developed form of cognitive behavioral therapy (CBT).The UP can be applied to a variety of disorders simultaneously and it can be easily performed in a group format. The UP was created on the basis of the identification of common psychopathological vulnerability factors in EDs [1, 10].In the UP, traditional CBT techniques (i.e., cognitive restructuring) are combined with more novel psychological skills (i.e., increasing awareness) to treat emotion regulation deficits, which are argued to be the underlying common factors in all EDs [11]. The UP is a structured, manual-based treatment [8, 9] which facilitates group delivery when patients present different EDs [11]. This, together with the fact that the UP can be applied simultaneously to individuals with different EDs, might help reduce existent waiting lists and current costs of individual treatment.

So far, studies exploring the effectiveness of the UP in a group format have led to promising findings [12–15].Overall, results suggest that the UP obtains medium-to-large effect sizes on numerous outcomes, including depression, anxiety, positive and negative affect, quality of life, overall adjustment, and avoidance of negative sensations, for both anxiety and mood disorders [12–14]. These studies have also revealed that between half and two thirds of patients ceased to meet diagnostic criteria after the treatment and one investigation, conducted by our team, revealed that changes remained stable 12 months after treatment completion [13]. Despite the previous results are encouraging, conclusions should be interpreted with caution as sample sizes have been small (11 participants in two studies and 47 patients in one investigation). Methodologically-sound, randomized, controlled trials are needed in order to replicate the aforementioned findings and to elucidate whether the UP in group format is indeed an effective treatment option for EDs in public settings.

The present non-inferiority, randomized, controlled trial will compare the efficacy of the UP in group format against traditional individual CBT treatment in a sample of patients with EDs. Ultimately, our goal is to explore whether the UP in group format can be an effective psychological intervention for EDs in the Spanish National Health System, that is, one that generates long-lasting changes in symptoms. To ensure the generalizability of results, our goal is tested in various public mental health centers in Spain.

## Methods/design

The current study is a non-inferiority, multicenter, randomized, controlled clinical trial with two treatment groups: UP in group format and traditional CBT in individual format (treatment as usual, TAU). A conservative, non-inferiority design was selected because, to the best of our knowledge, this is the first randomized controlled trial to compare the UP in a group format against individual CBT in public mental health settings.

**Fig. 1:**
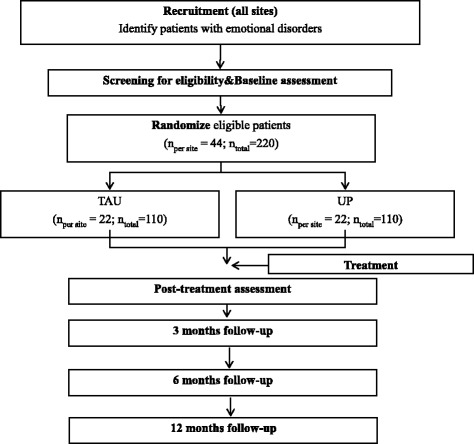
Study flow chart

**Fig. 2:**
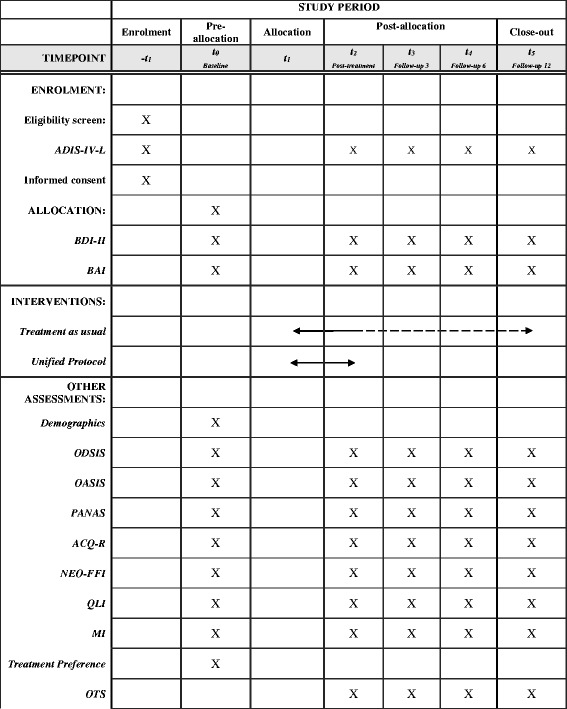
Study schedule of enrolment, interventions, and assessments

In the present investigation, all consecutive patients with EDs attending any of the collaborating centers (see “Sample and recruitment” section) are asked to participate. Once inclusion criteria are met (see “Inclusion and exclusion criteria” section), each patient is randomly assigned to one of the two experimental groups: TAU or UP (see “Procedure” section). The study includes 5 assessment points (baseline, post-treatment, and three follow-ups at 3, 6, and 12 months after treatment completion). The flow chart of study design is shown in Fig. 1. Also, a schedule of enrolment, interventions, and assessments is reported following the Standard Protocol Items: Recommendations for Interventional Trials (SPIRIT) guidelines (Fig. 2). Note that, despite treatment occurs at different frequencies between conditions (approximately once a month in the TAU condition and weekly in the UP group), assessment points are the same for both groups.

### Sample and recruitment

The trial started the recruitment in September 2014 and it is still active. The study is conducted in five different Public Mental Health Centers and Primary Care Centers in Spain, namely, the Elda Clinical General Hospital, the Hospital Clínico Universitario de Valencia, the Dr. Peset University Hospital, the PCC IIA Milagrosa of Pamplona, and the Hospital Comarcal de Vinaròs. Participants are individuals over 18 years of age seeking psychological assistance in the Spanish Public Health System. Sample size was set to 220 participants to ensure enough statistical power when testing our hypotheses (see “Sample size” section).

Patients are referred to the study by licensed psychologists, psychiatrists, and clinical psychology residents working at the collaborating centers, who also assess for current DSM diagnoses (See “measures” section) and the remaining eligibility criteria (see “Inclusion and exclusion criteria” section). Individuals with comorbid diagnosis of several EDs are also enrolled in the study. The existence of such comorbidities might be important for assessment scores. However, in the present study we did not stratify considering comorbidities because this could significantly increase the complexity of the design (i.e., some patients might have more than one comorbid diagnosis). Due to the difficulties derived from conducting an RCT in different public mental health settings, we cannot guarantee a 100% of independent evaluations. We expect between 60 to 75% of independent evaluations. The rest of evaluations will probably be made by recruiters who are also the therapists in one of the conditions. However, this information is collected and considered in the interpretation of results.

Patients meeting eligibility criteria are asked to participate and are provided with an information sheet, information on data confidentiality, and an informed consent form. After participation acceptance, patients complete the baseline assessment protocol in their correspondent health center (see “Measures” section). Next, participants are informed of the condition they have been randomly assigned to, that is, TAU or UP.

Enrollment period and waiting time are comparable across participants (2–3 weeks). When the time from assessment, diagnosis, and randomization to the onset of the intervention takes more than 2 weeks we establish a new period of assessment. For ethical reasons, if a patient feels uncomfortable with the group format at any time during the study, he/she is allowed to join the TAU condition. This information is collected and considered in the analyses.

### Sample size

To calculate the sample size required, the non-inferiority margin (i.e., tolerable amount of effect difference between TAU and the UP) has to be set. This was done according to the US Food and Drug Administration (FAD) [16] guidelines. First, the effect of the active control (i.e., CBT) compared with placebo needs to be established, based on previous research. Meta-analyses indicate medium-to-large effect sizes (between .4 and .8) of individual CBT on EDs [17–19]. Then, an amount of minimum preserved effect needs to be set. According to FDA guidelines, a preserved effect of a 50% of the effect of the active control (individual CBT) is recommended, so this was set to .3 in the present study. Because, to the best of our knowledge, no meta-analysis has reported effect sizes for each outcome included in the study, the margin has been defined according to the pooled estimate of overall effectiveness of CBT.

With regards to treatment satisfaction, a 35% non-inferiority margin was established. This is based on a meta-analysis of computer CBT, in which between 70% and 100% of depressed patients were satisfied with treatment [20]. To the best of our knowledge, there is no meta-analysis of treatment satisfaction with individual, face-to-face CBT and its study is rare. However, similar to group UP, computer CBT has been argued to be an alternative to traditional CBT [20], especially when resources are limited, so the aforementioned satisfaction rates of computer CBT might be relevant in the present study.

We obtained a sample size of 95 participants for each group with 80% power, an alpha level of .05 (one-sided), a standard deviation of .83 in the CBT group compared with placebo, and a non-inferiority limit of .30 [21, 22]. Considering a drop-out of 15%, we decided to recruit 110 subjects in each group. When registering the study, a sample size of 200 individuals was calculated. However, this was increased by 20 patients after considering non-inferiority sample size calculations.

### Procedure

Randomization has been performed by a researcher unrelated to the study using a computer-generated sequence (Randomizer). In the program, the researcher generated 1 set of numbers with 220 numbers, which had a 1-to-2 range. Randomization is stratified according to the severity of the primary measures of depression (BDI-II) [23, 24] and anxiety (BAI) [25, 26], using the recommended cut-off in manuals. In the BDI-II, these are 0–13 for normal, 14–19 for mild, 20–28 for moderate, and for 29–63: severe depressive symptoms [24]. As for anxiety symptoms (BAI), cut-off are 0–7 for normal, 10–18 for mild, 16–25 for moderate, and 26–63 for severe [26]. Stratification is made to ensure a comparable proportion of severely depressed and anxious individuals in each group. For each subgroup (i.e., severe or less severe depression and/or anxiety), participants are randomly assigned to the UP or to TAU. In the UP condition, there are between 8 and 10 participants and 2 clinicians (therapist and co-therapist) per group. In the TAU condition, participants receive individual sessions with one therapist.

### Eligibility criteria

Inclusion and exclusion criteria were set following the recommendations made by the authors of the UP [27] (Table 1).

**Table 1:** Eligibility criteria

Inclusion criteria	
1	Anxiety disorder, mood disorder, or adjustment disorder is the main diagnosis (most interfering and severe)^a^
2	The patient is over 18 years of age
3	The patient is fluent in the language in which therapy is performed (Spanish in the present study)
4	The patient is able to attend to the evaluation and treatment sessions and signs the informed consent form
5	Patients taking pharmacological treatment for their emotional disorder are asked to maintain the same dosages and medications for at least 3 months prior to enrolling in the study and during the whole treatment^b^
Exclusion criteria	
1	The patient presents a severe condition that would require to be prioritized for treatment, so that an interaction between both interventions cannot be ruled out. These include a severe mental disorder (bipolar disorder, schizophrenia, or an organic mental disorder),suicide risk at the time of assessment, or substance use in the last three months (excluding cannabis, coffee, and / or nicotine)
2	The patient has previously received 8 or more sessions of psychological treatment with clear and identifiable CBT principles within the past 5 years.

^a^The following disorders will be included based on DSM-IV-TR diagnostic criteria [21]: major depression disorder, dysthymic disorder, panic disorder, agoraphobia, obsessive-compulsive disorder, generalized anxiety disorder, posttraumatic stress disorder, social anxiety disorder, hypochondria, and adjustment disorders. Patients with unspecified anxiety disorders and unspecified depressive disorders will also be included as they are frequent in public settings

^b^If medication stability is not possible, the participant’s data will be treated separately in the analyses

### Ethics

This study is carried out in accordance with the study protocol, the Helsinki Declaration, and good clinical practice. This non-inferiority, multicenter, randomized, controlled clinical trial has been approved by ethical and research committees of all collaborating centers: Hospital General Clínico de Elda (N°. PUG-02-14), Hospital Clínico Universitario de Valencia (N°. F-CE-GEva-15), Hospital Universitario Dr. Peset (N°. CEIC 53/15), CSM IIA Milagrosa (N°. Pyto2016/41), and Hospital Comarcal de Vinaròs (N°. 0103–2016). Data handling is carried out according to premises established by Spanish laws [28]. Security and confidentiality of participants’ data are guaranteed by using alphanumeric codes (SUP001) instead of names.

### Therapists and interventions

Therapists participating in the study include licensed psychologists with between 8 and 20 years of experience in delivering CBT, clinical psychology residents with 2 to 4 years of experience, and doctoral students in clinical psychology with between 3 and 5 years of experience.

### Unified protocol in group format

Our research team has adapted the UP [8, 9] to implement it in group format in a public mental health setting in Spain. This adaptation is composed of 12, two-hour treatment sessions, at a rate of one session per week. Treatment content, split by session, is shown in Table 2.To encourage maximum fidelity to the protocol, all therapists participating in this condition received a UP training course prior to study onset, as well as videoconference supervision before every session. The course consisted of a 20-h group workshop divided in 3 sessions. Next, all therapists received an individual training during 12 therapy sessions. Depending on availability, this individual training consisted of an online supervision before sessions or it involved acting as a co-therapist. Supervision is conducted by the leading author, J.O., who has been certified UP Researcher/Trainer by the Unified Protocol Institute.

**Table 2:** Treatment content split by session

Session number	Content
Session1	Motivation for change and commitment to treatment
Session2	Understanding the adaptability of emotions
Session3	Recognition and analysis of emotions
Session4	Emotional awareness training - I
Session5	Emotional awareness training - II
Session6	Cognitive flexibility- I
Session7	Cognitive flexibility- II
Session8	Emotional avoidance and emotion-driven behaviors
Session9	Consciousness and tolerance to physical sensations
Session10	Interoceptive and situational emotional exposure- I
Session11	Interoceptive and situational emotional exposure- II
Session12	Achievements, maintenance, and relapse prevention

Participants who miss one or more UP sessions receive a phone call from the group therapist to explore the reasons for non-attendance, as well as to encourage reading the materials that summarize missed sessions. Also, at the beginning of each session the therapist reviews important contents of the past session to minimize this effect of missed sessions.

### Treatment as usual (TAU)

Individual CBT is the treatment of choice by psychologists and psychiatrists at the collaborating Public Mental Health Centers and Primary Care Centers, together with pharmacological treatment (i.e., antidepressants and / or anxiolytics). Clinicians in this condition complete a self-report sheet describing the characteristics of their interventions with treatment modules as cues (psychoeducation module, identification of negative thoughts, breathing training, etc.), the average duration of sessions, number of sessions delivered, and end-of-treatment date, as well as information on the number of appointments with the psychiatrist and pharmacological treatment prescribed during the study. Information on the number of appointments with the psychiatrist and pharmacological treatment prescribed during the study is also collected for patients in the UP group following the same procedure described for the CBT condition.

As opposed to the UP condition, in which the duration of treatment is fixed (12 sessions), the usual practice in Spanish public settings is that treatment ends when both the clinician and the patient agree that the intervention has been effective. This is not necessarily guided by questionnaires or a diagnostic interview, but on clinical judgment. Because the study goal is to compare a new treatment (UP in a group format) with the usual practice in Spanish public settings, the same procedure described above will be used to determine the end of treatment in the TAU condition and no specific end points (i.e., a decrease in questionnaire scores) or number of sessions will be imposed. Because this is likely to yield different treatment durations between study conditions, the treatment duration (number of sessions) will be used as a covariate in the analyses.

### Measures

The evaluation protocol is administered by therapists in a paper and pencil format at the participant’s health center in 5 different times: baseline, post-treatment, and follow-ups at 3, 6, and 12 months. To minimize biases, every administration of the diagnostic interview is done by two different psychologists, one face-to-face and the other by phone. Inter-rater reliability will be calculated. Assessment instruments include demographic characteristics (age, sex, education, marital status, and work status), a diagnostic interview, and well-established questionnaires for both primary and secondary outcomes.

### Primary outcomes

Severity of depression and anxiety symptoms are assessed with the Beck Depression Inventory (BDI-II) [23, 24] and the Beck Anxiety Inventory (BAI) [25, 26], respectively.

In addition to symptoms, a current diagnosis of anxiety and/or mood disorder is made with the lifetime version of the Anxiety Disorders Interview Schedule for DSM-IV (ADIS-IV-L) [4, 29]. The ADIS-IV-L is a semi-structured interview based on the diagnostic criteria of the Diagnostic and Statistical Manual of Mental Disorders -4th ed. (DSM-IV-TR) [4]. We have not used the ADIS-5 because it is not available in Spanish yet.

### Secondary outcomes

We administer the Overall Depression Severity and Impairment Scale (ODSIS) [30] and the Overall Anxiety Severity and Impairment Scale (OASIS) [31] weekly to assess the severity of depressive and anxiety symptoms, respectively. These measures are included to complement the results obtained with the BDI-II and the BAI, as well as to obtain a continuous assessment. The Positive and Negative Affect Scale (PANAS) [32, 33] is administered to evaluate positive and negative affect. The perception of control over anxiety is assessed by means of the Anxiety Control Questionnaire-Revised (ACQ-R) [34, 35]. Personality is measured with the NEO Five-Factor Inventory (NEO-FFI) [36], which offers a rapid and general measure of the Big Five personality traits: Neuroticism, Extraversion, Openness to experience, Agreeableness, and Conscientiousness. The Quality of Life Index (QLI) [37] is used to evaluate several aspects of health-related quality of life (i.e., physical disability, emotional well-being, self-care and independent functioning, occupational functioning, interpersonal functioning, social emotional support, community and services support, personal fulfillment, spiritual fulfillment, and overall quality of life). Similarly, the Maladjustment Inventory (MI) [38] is used to evaluate the extent to which the subject’s current problems impact negatively on different areas of daily life, namely, work, social life, leisure time, relationship with the partner, family life, and overall adjustment in daily activities. Additionally, we created an ad hoc questionnaire, the Treatment Opinion Scale (OTS), which is administered to participants in both conditions. Our ad hoc questionnaire evaluates the quality of the intervention (i.e., “How would you rate the quality of the treatment program you have received?”) and that of its components (i.e., “Have the techniques and exercises that we have practiced helped you regulate your emotions properly?”). Also, it measures the amount of discomfort experienced during treatment (i.e., “To what extent has this treatment caused you discomfort?”) and the experience of participating in a group format (i.e., “If you were to seek help again, would you choose a group treatment program?”). Some questions in the OTS use a4-point Likert scale (0 = “*poor”* or “*nothing”* to 3 = “*excellent”* or “*very much”*), while an 11-point response scale is selected in some items (0 = “*nothing”* to 10 = “*very much”*).

All measures used in the study have been standardized in Spanish. Administration time is between 90 and120 minutes for the ADIS-IV-L and approximately 90 min for the primary and secondary outcomes altogether.

### Analyses

First, we will calculate between-group differences in baseline measures, to ensure that randomization resulted in comparable groups. When normally distributed, continuous data will be analyzed via *t* test. If the Kolmogorov-Smirnov test indicates a non-normal distribution, a Mann-Whitney *U* test will be preferred. A chi-square test will be performed for categorical data. Then, to compare the efficacy of treatments, we will use a mixed-effects regression modeling with full information maximum likely hood estimation. This method is recommended over repeated-measures ANOVAs due to its flexibility in handling missing data [39]. Group (TAU or UP) will be used as the between-subject factor and time (baseline, post-treatment, and follow-ups) as the within-subject factor. Standardized effect sizes (Cohen’s *d*) will be calculated both for within and between-group analyses. Covariates will be demographic and clinical variables that are not comparable between groups, as exposed in the beginning of this section. Effect estimators in the UP group will be adjusted to control for the impact of group dependencies (i.e., homogenization of response to treatment due to shared environment), following the recommendations of Baldwin et al. [40].

To address problems of missing data or premature dropouts, an intention-to-treat analysis will be used and the most recent data will be imputed (last observation carried forward approach). This analytic strategy will only be performed with patients attending at least 50% of sessions, as recommended in past research [41], and with those responding to at least one of the post-treatment assessments (i.e., end of treatment or follow-ups) so that treatment effect may be captured by the last observation. In addition to the last observation forward, missing data in the UP group will also be replaced with the CBT mean +/− the non-inferiority margin and missing data in the CBT group will be replaced with the UP mean (i.e., bias toward inferiority) [42]. Results using a last observation carried forward approach and a bias toward inferiority imputation will be compared against a completers-only strategy and all of them will be reported. To ensure non-inferiority, a per-protocol analysis will also be conducted. Following FDA guidelines [16], non-inferiority will be concluded if the upper bound of the 95% CI for the effect estimate in the UP condition is smaller than the non-inferiority margin (50% of the effect estimate of individual CBT when compared to placebo). Due to the non-inferiority design (i.e., the Unified Protocol in a group format is not worse than individual CBT), a one-sided confidence interval will be used in the analyses. A post-hoc power analysis with the primary outcomes will be conducted at post-treatment and follow-ups using the sample size and obtained effect size.

The researcher analyzing data, C.S.R., will be blind to arm allocation. To ensure that, arms will be given arbitrary letters (A and B) in the database and only the lead researcher, J.O., will know the correspondence between study allocation arms and assigned arbitrary letters.

Reporting of results will follow the Consolidated Standards of Reporting Trials (CONSORT) recommendations [43].

## Discussion

EDs have become a matter of public concern due to their high prevalence and associated costs [6, 7, 44]. The UP has emerged as a promising form of CBT to be used across diagnostic categories both in individual and group format [8, 9]. Therefore, the study of a therapeutic approach that can be administered to several patients with different disorders simultaneously, as in the present study, may have important clinical implications for countries.

The use of the UP in group format in public mental health settings may have some advantages over traditional individual CBT: 1) it may allow clinicians to use the same treatment for a variety of psychological disorders [8, 9, 12]; 2) it might help reduce existing waiting lists as between 6 and 8 patients can be treated simultaneously, which would probably reduce the duration of suffering in unattended patients; 3) in contexts similar to the Spanish public system, where individual CBT is the norm and therapy sessions occur at long intervals (i.e., once a month) due to waiting lists, a UP-based treatment program in group format can help increase the frequency of sessions because more patients are treated simultaneously, which may facilitate a more rapid detection of patients’ problems during treatment; 4) finally, group therapy is known to report benefits for the patient that are not obtained with individual treatment, such as reducing isolation, facilitating social support, and learning from others’ experiences [45, 46], which might enhance its efficiency. The present RCT taps into the aforementioned aspects of treatment satisfaction and effectiveness.

Our study has also some limitations. First, some people who ask for treatment in the public mental health system prefer individual treatment, which could be a barrier when randomly assigned to a group format (i.e., dropout or decreased satisfaction and efficacy of the UP). To control for this, we will explore the effect of format preference and format assignment on treatment adherence and effectiveness. Additionally, as conditions differ in terms of type of therapy (CBT vs. UP) and format (individual vs. group), it will be difficult to ascertain what was responsible for the results. Despite the present study only seeks to find whether the UP in a group format can be an alternative to the traditional psychological treatment in public settings in Spain (i.e., individual CBT), results should be interpreted with caution and no conclusions can be drawn on whether results are due to differences in the type of therapy, its format, or both. In line with the previous, it is important to note that the usual treatment was not stabilized across clinicians at centers (i.e., duration of sessions and treatment) precisely to ensure that the newly tested treatment, namely the UP in a group format, was compared against the usual treatment in Public Mental Health Settings in Spain. It is also important to note that, due the multicenter nature of the study, interventions are conducted by different clinicians, so implementation could differ across centers. In order to minimize this effect, a certified UP Researcher/Trainer, J.O., has been in charge of the full treatment for the first two groups at all collaborating centers. All clinicians will act as co-therapists of one group at their working center. Also, when they start treatment they will receive a videoconference supervision before every session by J.O. Despite this is done to promote fidelity to the protocol, it is important to note that assessment of fidelity is not performed by an independent rater, so true fidelity cannot be ensured. Finally, another shortcoming is that interviewers using the ADIS are not blind to treatment arm. This was not possible due to legal reasons (data collection at participating hospitals had to be done by a psychologist/psychiatrist working at the center). Due to time restrictions of psychiatrists, this was done by psychologists, who were also in charge of treatment. Despite this limitation, it is important to note that the ADIS is an objective, structured interview, and that on each assessment two interviews are independently made by two different psychologists, which should reduce the likelihood of biases.

Despite the aforementioned shortcomings, the present study may have important clinical implications as it is the first randomized, controlled clinical trial to test the effectiveness of a transdiagnostic intervention in group format for the treatment of EDs in public settings in Spain. Results will reveal whether the use of the UP in group format may serve to reduce existent waiting lists without decreasing the effectiveness of interventions (non-inferior to TAU).This would have important implications for patients, as it would possibly allow treatments to be more intensive (i.e., on a weekly basis instead of monthly) thanks to the simultaneous treatment of an increased number of patients.

## Abbreviations

A: Agoraphobia
ACQ-R: Anxiety Control Questionnaire-Revised
AD: Adjustment Disorder
ADIS: Anxiety Disorders Interview Schedule
ADNOS: Anxiety disorder not otherwise specified
BAI: Beck Anxiety Inventory
BDI: Beck Depression Inventory
DD: Dysthymic disorder
DSM-IV-TR: Diagnostic and statistical manual of mental disorders, fourth text-revised edition
EDs: Emotional Disorder
GAD: Generalized anxiety disorder
H: Hypochondria
IA: Inventory for Agoraphobia
LQI: Life Quality Index
MDD: Major depression disorder
MDNOS: Mood Disorder Not Otherwise Specified
MI: Maladjustment Inventory
NEO-FFI: NEO-Five Factor Inventory
OASIS: Overall Anxiety Severity and Impairment Scale
OCD: Obsessive-Compulsive Disorder
ODSIS: Overall Depression Severity and Impairment Scale
OTS: Opinion of Treatment Scale
PANAS: Positive and Negative Affect Schedule– Negative Affectivity
PCC: Primary Care Center
PD: Panic Disorder
PMHC: Public Mental Health Center
POST-T: Post-treatment
RCT: Randomized Controlled Trial
SAD: Social Anxiety Disorder
TAU: Treatment As Usual
UP: Unified Protocol
